# High Geriatric Nutritional Risk Index Risk as a Predictor of Postoperative Complications and Early Mortality in Older Adult Patients Undergoing Pancreatoduodenectomy for Periampullary Malignancies

**DOI:** 10.3390/jcm14020655

**Published:** 2025-01-20

**Authors:** Ming-Hung Wang, Chien-Yu Chen, Yu-Hung Lin, Yueh-Wei Liu, Yu-Yin Liu, Wei-Feng Li, Chang-Ting Lin, Szu-Wei Huang, Cheng-Hsi Yeh, Shih-Min Yin

**Affiliations:** 1 Division of General Surgery, Department of Surgery, Kaohsiung Chang Gung Memorial Hospital and Chang Gung University College of Medicine, 123, Dapi Road, Niaosong District, Kaohsiung 833, Taiwan; rfv123@cgmh.org.tw (M.-H.W.); a0978337257@cgmh.org.tw (C.-Y.C.); adrianlin107@cgmh.org.tw (Y.-H.L.); anthony0612@cgmh.org.tw (Y.-W.L.); liuyuyin@cgmh.org.tw (Y.-Y.L.); webphone@cgmh.org.tw (W.-F.L.); ycc9002108@cgmh.org.tw (C.-H.Y.); 2 Weight Management Center, Kaohsiung Chang Gung Memorial Hospital and Chang Gung University College of Medicine, Kaohsiung 833, Taiwan; 3 Division of Hematology Oncology, Department of Internal Medicine, Kaohsiung Chang Gung Memorial Hospital and Chang Gung University College of Medicine, Kaohsiung 833, Taiwan; linctin05@cgmh.org.tw; 4 Department of Obstetrics and Gynecology, Kaohsiung Chang Gung Memorial Hospital and Chang Gung University College of Medicine, Kaohsiung 833, Taiwan; b9602067@cgmh.org.tw

**Keywords:** pancreaticoduodenectomy, old age, malnutrition, Geriatric Nutritional Risk Index, postoperative outcome

## Abstract

**Introduction:** Pancreaticoduodenectomy (PD) is a major surgery associated with significant morbidity and mortality, especially in older adult patients. Malnutrition is a common complication in these patients and is linked to poorer outcomes. This study aimed to investigate the associations between preoperative nutritional status using the Geriatric Nutritional Risk Index (GNRI) and postoperative outcomes in older adult patients who underwent PD. **Methods:** A retrospective cohort study was conducted on 363 older adult patients who underwent PD. The preoperative GNRI was calculated based on serum albumin levels and body mass index. GNRI ≤ 82, GNRI 83 to ≤98, and GNRI > 98 were classified as severely malnourished, moderately/mildly malnourished, and no malnourishment, respectively. Perioperative data, including demographics, comorbidities, and postoperative complications, were collected. Univariate and multivariate analyses were performed to assess the associations between the GNRI and outcomes such as length of hospital stay, postoperative complications, and overall survival. **Results:** Patients with a higher GNRI were more likely to experience Clavien–Dindo grade ≥ 3b postoperative complications (42.1% vs. 22.0% vs. 14.1%; *p* = 0.027) and pulmonary complications (26.3% vs. 11.9% vs. 4.2%; *p* = 0.016). These patients also stayed at the hospital for a longer duration (17.0% vs. 16.0% vs. 11.0%; *p* < 0.001). Multivariate analysis confirmed that the GNRI was an independent predictor of adverse outcomes, even after adjusting for other confounding factors. **Conclusions:** Our findings highlight the importance of preoperative nutritional assessment in older adult patients undergoing PD. Patients with low GNRI scores are at increased risk of postoperative complications and prolonged recovery. These results underscore the need for targeted nutritional interventions and regular monitoring of these patients. Future studies should focus on interventions to improve nutritional status in older adult patients undergoing PD.

## 1. Introduction

Pancreaticoduodenectomy (PD) is the standard surgical intervention for pancreatic cancer and malignant tumors of periampullary regions [1]. With an increase in access to healthcare facilities, the number of older adult patients undergoing major surgeries like PD is increasing. Although advancements in surgical techniques, instrumentation, and perioperative care have improved overall outcomes [2,3], the mortality and morbidity rates following PD in the older adult population are still significantly high [4,5].

Malnutrition is frequently observed in patients with malignant tumors, leading to compromised immunologic status and heightened inflammatory activity [6,7,8]. It is reported that malnutrition presents in 20–70% of cancer patients depending on the feature of the cancer and the clinical stage [9,10]. Malnutrition is a known significant risk factor for adverse postoperative outcomes. Numerous studies have demonstrated that preoperative malnutrition increases the risk of postoperative complications and negatively impacts long-term survival [11,12,13,14]. In addition to surgery, there are several other options for cancer treatment. For example, patients with pancreatic cancer who are borderline resectable or have locally advanced disease may undergo neoadjuvant chemotherapy. We aim to discuss whether malnutrition in patients is associated with a condition of being borderline resectable. Furthermore, neoadjuvant chemotherapy in periampullary malignancies is associated with a decline in nutritional parameters, including rapid turnover proteins, as well as an increase in inflammation-related prognostic scores. Consequently, these nutritional changes can complicate recovery and reduce chemotherapy tolerance, ultimately affecting treatment success [15]. Therefore, early nutritional intervention is crucial for optimizing outcomes and supporting patient resilience during treatment [16]. However, related studies focus on how postoperative malnutrition influences the early postoperative outcomes and survival of older adult patients who have PD for periampullary cancer were limited.

Several clinical tools are available to assess the nutritional statuses of older adult patients, such as Nutritional Risk Screening 2002, the Mini Nutritional Assessment (MNA), and the Subjective Global Assessment (SGA) [17,18]. However, these tools gather data based on the clinical history and physical examination of patients, which can introduce bias, complicate retrospective analysis, and be time-consuming. The Geriatric Nutritional Risk Index (GNRI), which is calculated using serum albumin and the body mass index (BMI), was developed to be used as a practical and objective measure of nutritional status [19]. Recent studies have underscored a significant correlation between the GNRI and postoperative recovery in elderly patients undergoing major abdominal surgery [14,20], as well as patients with cancer prognosis [21,22,23], acute respiratory distress syndrome (ARDS) [24,25], infections, and trauma [26,27].

## 2. Materials and Methods

### 2.1. Patient Selection Criteria and Nutritional Status Evaluation

**Figure 1:**
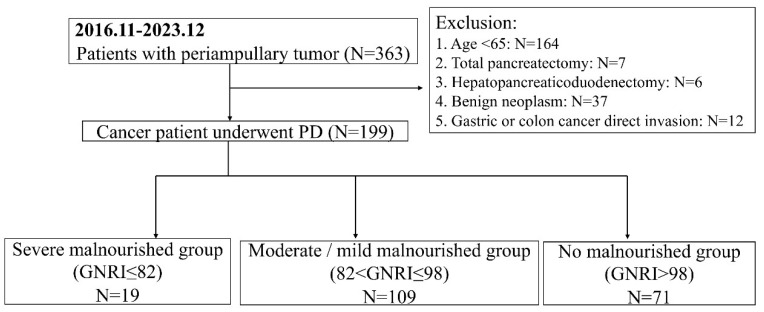
A flow chart of the patient selection process. PD, pancreaticoduodenectomy; GNRI, Geriatric Nutritional Risk Index.

A total of 363 patients who had received PD for periampullary neoplasm at Kaohsiung Chang Gung Memorial Hospital were retrospectively enrolled in this study, covering the period from November 2016 to December 2023. Patients were excluded if they had benign neoplasms confirmed by pathology, were <65 years of age, underwent total pancreatectomy or hepatopancreaticoduodenectomy for oncologic reasons, or had pancreaticoduodenectomy owing to direct invasion from gastric or colon cancer. A flow chart with the patient selection process is shown in Figure 1.

The open surgical approach was recommended to patients with advanced pancreatic adenocarcinoma with consideration of possible vascular reconstruction. There were no other contraindications for minimally invasive techniques. The methods for performing both traditional open PD and minimally invasive PD, which encompass laparoscopic and robotic techniques, have been outlined in earlier descriptions [2].

Preoperative nutritional status was assessed using the GNRI within 30 days prior to surgery based on the data closest to the surgical date. We retrospectively collected key nutritional parameters including height, weight, BMI, and serum albumin levels. The GNRI was determined using the following formula: GNRI = 1.489 × serum albumin level (g/L) + [41.7 × (actual body weight/ideal body weight (IBW))]. According to Bouillanne et al.’s original approach, if the ratio of actual body weight to IBW was ≥1, it was capped at 1. Participants were categorized into three groups adapted from Bouillanne et al.’s four-class model: severely malnourished (GNRI < 82), moderately/mildly malnourished (GNRI 82 to ≤98), and no malnourishment (GNRI > 98).

### 2.2. Perioperative Patient Management Protocol

The perioperative and postoperative management and nutrition support strategy is described below. Patients who had significant drops in body weight > 5% in the past 3 months, BMI < 18.5, or hypoalbuminemia < 3.5 g/dL were offered a complimentary nutritional intervention by a dietitian, along with preoperative enteral or parenteral nutritional support. Patients were also advised to participate in preoperative rehabilitation programs and respiratory training. Biliary decompression was scheduled for patients who had total bilirubin levels exceeding 10 mg/dL, showed evidence of biliary tract infection, or underwent neoadjuvant chemotherapy. To prevent venous thromboembolism, mechanical approaches like compression stockings and intermittent pneumatic compression were used instead of chemical thromboprophylaxis. Nasogastric tubes, Foley catheters, and open drains were placed in all patients to enable the early detection of postoperative bleeding and leaks.

The nasogastric tube was typically removed on postoperative day 3, following which patients were allowed to sip water following the passage of flatus. The diet was gradually advanced based on patients’ appetite. Routine artificial nutritional support was administered for 1 week after surgery, with extended use if there was delayed gastric emptying (DGE), ileus, an anastomotic leak delayed enteral feeding, or oral intake failure to reach 60% of daily requirements after postoperative day 7. All patients received somatostatin analogs at least 3 days following surgery, and this could be extended to a week based on the postoperative pancreatic fistula (POPF) status. All patients received early ambulation and rehabilitation on postoperative day 1, and the urinary catheter was removed once the patient was able to ambulate effectively. All of the surgical drains were removed before discharge, and most of the patients discharged within 4 weeks after surgery.

### 2.3. Postoperative Early Postoperative Outcomes and Survival Evaluation

The primary outcomes of our study were in-hospital mortality and morbidity. Additional surgical outcome evaluations included intraoperative estimated blood loss, total operative time, the duration of postoperative hospital stay, the duration of intensive care unit (ICU) stay, time to first oral soft diet, ambulation initiation, the duration of total parenteral nutrition (TPN) use, and the length of surgical drain placement. Postoperative complications documented included DGE, POPF, postpancreatectomy hemorrhage (PPH), surgical site infection (SSI), and postoperative pulmonary complications (PPCs). POPF was classified according to the 2016 guidelines of the International Study Group on Pancreatic Fistula [28]. SSI was defined according to the criteria established by the United States Centers for Disease Control and Prevention [29]. PPCs are defined as any adverse events affecting the respiratory system following anesthesia and surgery, including pneumonia, aspiration pneumonitis, pleural effusion, ARDS, and others [30]. Postoperative complications were categorized according to the Clavien–Dindo classification system, and mortality referred to patient fatality occurring in the initial hospitalization after surgery.

In our investigation of predictors for early postoperative surgical outcomes, we specifically concentrated on significant postoperative complications (characterized as Clavien–Dindo grade ≥ IIIb), PPCs, extended hospital stays (defined as longer than 28 days), and mortality within 1 year. The difference in the GNRI between operations was used for evaluating the perioperative nutrition change. Using the formula ΔGNRI% = (preoperative GNRI − postoperative GNRI)/preoperative GNRI × 100%, we defined the cut-off point for the ΔGNRI% as 4.5% for analyzing the postoperative complications and early postoperative surgical outcomes. Both univariable and multivariable analysis were conducted to evaluate these outcomes, adjusting for potential confounders. The median operation time was 469 min. We used 480 min as the cut-off point for univariable and multivariable analyses. This approach allowed us to comprehensively evaluate the effects of the GNRI and other relevant predictors on early postoperative surgical outcomes in this patient population.

### 2.4. Postoperative Long-Term Overall Survival Evaluation

The secondary outcome of our study was long-term postoperative mortality. Overall survival (OS) was measured as the period between the date of resection and the date of last visit to the outpatient center or death from any cause. As most of the patients included in the study group had pancreatic cancer with a relatively poor prognosis, we separately analyzed OS based on GNRI risk in pancreatic cancer and other malignancies.

### 2.5. Statistical Analyses

Data analyses were performed using IBM SPSS Statistics for Windows, version 25.0 and NCSS 2024 software. Pearson’s chi-square tests were conducted for categorical variables. The Kolmogorov–Smirnov test was employed to assess the normality of distribution for continuous variables. Student’s *t*-tests were used to compare means for continuous variables with normally distributed ones, while Mann–Whitney U-tests were employed for those non-normally distributed ones. To address potential confounding factors, logistic regression analyses were performed at both the univariable and multivariable levels. Survival distribution among the grouped patients was assessed using the Kaplan–Meier method, with differences between the curves analyzed through the log-rank test. All tests were two-sided, and statistical significance was set at a *p*-value < 0.05.

## 3. Results

### 3.1. Comparison of Patient Characteristics Based on GNRI Categories

A total of 199 older adult patients who underwent PD were stratified according to the GNRI categories. In this cohort, 19 were severely malnourished patients, 109 were moderately/mildly malnourished patients, and 71 were no malnourishment patients. The demographics and patient characteristics are demonstrated in Table 1. The severe malnourished group consisted of older patients, who had lower BMI values and poorer performance statuses than those in the other groups. Median age in the severely malnourished group was 76 years, which was significantly higher than the no malnourishment group (71 years, *p* = 0.006). The BMI was markedly lower in the severely malnourished group (median 19.01 kg/m²) compared with the moderately/mildly malnourished (22.78 kg/m²) and no malnourishment groups (24.14 kg/m², *p* < 0.001). In terms of performance status, 31.6% of the severely malnourished group had an ECOG score of 1, which was significantly higher than the moderately/mildly malnourished (7.3%) and no malnourishment groups (2.8%, *p* = 0.002). The prevalence of hypertension and diabetes mellitus was significantly lower in the severely malnourished group compared with the other two groups. Other baseline characteristics including a history of abdominal surgery, medical comorbidity, tumor type, and surgical approach were not statistically significantly different in the groups.

### 3.2. Impact of GNRI Categories on Early Postoperative Outcomes and Postoperative Morbidity or Mortality

The unadjusted early postoperative outcomes based on GNRI-defined malnutrition categories are summarized in Table 2. Patients in the severely malnourished group experienced a remarkably longer operative time (*p* = 0.023), a longer duration of postoperative hospital stay (*p* < 0.001), and delayed ambulation (*p* = 0.044). There was a significant difference in the severely malnourished group compared with moderately/mildly nourished and no malnourishment groups (*p* < 0.001). The average size of the tumor was 3.0 ± 1.5 cm, with sizes in the severely, moderately/mildly, and no malnourishment groups being 3.4 ± 1.4 cm, 2.9 ± 1.5 cm, and 3.3 ± 1.6 cm, respectively. There was a significant difference between the moderate/mild malnourishment group and the other groups (*p* = 0.024), but no significant difference between the severe and no malnourishment groups. Additionally, incidences of Grade 3b complications were significantly higher in the severely malnourished group (42.1%) compared with the moderately/mildly malnourished (22.0%) and no malnourishment groups (14.1%) (*p* = 0.027). PPCs were more frequent in the severe malnourished group (26.3%) compared with the no malnourished group (4.2%) (*p* = 0.016). Although no significant differences were observed in the early postoperative mortality, the 1-year mortality rate was notably higher in the severely malnourished group (78.9%) compared with the no malnourishment group (40.8%) (*p* = 0.011). There were no statistically significant differences among the groups in terms of ICU stay duration, TPN use, SSI, POPF rates, postoperative bleeding, or delayed gastric emptying.

Using the ΔGNRI% for analyzing postoperative complications and early postoperative surgical outcomes, only TPN use (*p* = 0.003) and postoperative pulmonary complication (*p* = 0.045) had significant differences. There were no statistically significant differences among the groups in terms of operative time, estimated blood loss, tumor size, duration of ICU stay, soft diet, ambulation, drain remove days, incidence of Grade 3b complications, SSI, POPF rates, postoperative bleeding, or delayed gastric emptying (Supplementary Table S1).

The univariate and multivariate analyses for factors associated with significant postoperative complications, PPCs, and extended hospital stays are presented in Table 3, Table 4 and Table 5. Table 3 presents the significant factors associated with significant postoperative complications (Clavien–Dindo grade ≥ IIIb). Severe malnourishment (odds ratio (OR) 4.43, *p* = 0.010), American Society of Anesthesiologists (ASA) physical status III–V (OR 4.18, *p* = 0.010), operative time > 480 min (OR 2.02, *p* = 0.048), and estimated blood loss > 400 mL (OR 2.05, *p* = 0.046) were associated with increased risk of major complications in univariate analysis. After adjustment in multivariate analysis, ASA physical status III–V (OR 3.56, *p* = 0.025) and severe malnourishment (OR 3.44, *p* = 0.040) remained significant predictors. For pulmonary complications (Table 4), univariate analysis showed that older age (OR 1.09, *p* = 0.038), male sex (OR 3.55, *p* = 0.028), and severe malnourishment (OR 8.10, *p* = 0.008) were significant predictors of PPCs. After multivariate adjustment, male sex (OR 4.40, *p* = 0.013) and severe malnourishment (OR 5.91, *p* = 0.034) remained significant (Table 4). For the extended hospital stays after surgery (>28 days) analysis (Table 5), the univariate analysis showed age (OR 1.06, *p* = 0.035), moderate/mild malnourishment (OR 2.01, *p* = 0.033), severe malnourishment (OR 4.37, *p* = 0.007), and minimally invasive surgery (MIS) (OR 0.45, *p* = 0.040) to be significant predictors. After multivariate adjustment, severe malnourishment (OR 3.54, *p* = 0.024), moderate/mild malnourishment (OR 1.96, *p* = 0.047), and the MIS approach used (OR 0.42, *p* = 0.030) remained significant (Table 5).

For analysis of predictors for postoperative 1-year early mortality (Table 6), univariate analysis demonstrated severe malnourishment (OR 5.43, 95% CI 1.64–18.04, *p* = 0.006), lower BMI (OR 1.1, 95% CI 1.01–1.19, *p* = 0.022) and no diabetes status (OR 2.04, 95% CI 1.14–3.70, *p* = 0.022) to be associated with higher early mortality. After multivariate adjustment, severe malnourishment only significantly predicted worse outcomes (adjusted OR 3.65, 95% CI 1.01–13.13, *p* = 0.048).

### 3.3. Impact of GNRI Categories on Long-Term Overall Survival

**Figure 2:**
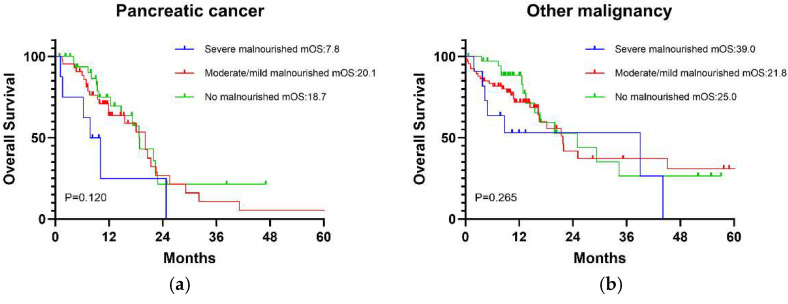
Long-term overall survival based on GNRI-defined malnutrition categories. GNRI, Geriatric nutritional risk index. (**a**) Pancreatic cancer. (**b**) Other malignancy.

Long-term OS curves in patients based on GNRI-defined malnutrition categories are summarized in Figure 2. The median OS months following pancreatoduodenectomy in patients with pancreatic cancer in the GNRI severely malnourished, moderately/mildly malnourished, and no malnourishment groups were 7.8, 20.1, and 18.7 (Figure 2a). The median OS months following pancreatoduodenectomy in other malignancy patients in the GNRI severely malnourished, moderately/mildly malnourished, and no malnourishment groups were 39, 21.8, and 25.0 (Figure 2b). When identified as a prognosis variable, GNRI severe malnourishment did not predict OS in pancreatic cancer patients following pancreatoduodenectomy (*p* = 0.120). Similarly, the OS rate was not significantly different between other malignancy patients with or without GNRI severe malnourishment (*p* = 0.265).

## 4. Discussion

Older adults are at a higher risk for nutritional deficiency, often due to various challenges associated with aging, including limitations in mobility, mental health issues, and changes in bodily functions [31,32]. Older adult patients with ampullary cancer are especially vulnerable to malnutrition owing to mechanical obstruction of oral intake and digestive impairment resulting from the local and systemic effects of the disease [33,34]. Following PD, a compromised digestive function in these patients often causes long-term negative impacts on patient clinical outcomes and nutritional status [12,35]. Consequently, these factors can reduce the likelihood of subsequent adjuvant therapy and negatively impact overall survival. Yet, research on whether postoperative malnutrition influences early postoperative outcomes and survival in older adult patients who have undergone PD for ampullary carcinoma is limited.

This study showed that a lower GNRI score is associated with longer operative time. We speculated that severe malnutrition significantly complicates surgical procedures by adversely affecting tissue integrity and healing capacity. Malnourished patients often exhibit compromised protein synthesis, leading to weakened tissue structures [36]. Furthermore, malnutrition can lead to alterations in fluid balance, potentially causing tissue edema [37]. Edematous tissues are more challenging to handle during surgery due to their increased friability and the obscured anatomical planes, which can prolong operative times and elevate the risk of inadvertent damage to surrounding structures.

Significant associations between lower preoperative GNRI and increased incidences of serious complications, PPCs, and prolonged recovery following surgery were noted. Multivariate analysis demonstrated an independent association between a lower GNRI score and adverse outcomes after adjusting for confounding factors such as age, ASA status, operation time, and estimated blood loss. These findings align with previous research that indicated malnutrition was linked to poorer early postoperative outcomes after pancreatic resection such as SSI [38], elevated risk of POPF [39,40], and other morbidities [41]. Our study provided significant evidence about severe malnourished GNRI in older adult patients with periampullary malignancies. Given the high complication rates and delayed postoperative recovery observed in patients in the severe malnourished GNRI group, aggressive preoperative nutritional support or postponing surgery in favor of alternative treatments could be considered an option before taking a surgical approach.

Notably, the present study showed a significant association between severe malnourished and the occurrence of PPCs. This finding is consistent with the existing literature that highlights the impact of malnourishment status to PPCs. Yu et al. reported that an inferior Prognostic Nutritional Index (PNI) was associated with a higher incidence of PPCs after radical cystectomy [42]. Preoperative controlling of nutritional status score is an independent predictor of PPCs and 1-year mortality in patients with resectable non-small-cell lung cancer [43]. In our study, PPCs mostly presented as aspiration pneumonia, which frequently occurs in older adult patients owing to weakened respiratory muscles, diminished cough reflex, and impaired immune function, making it difficult to clear secretions and protect against aspiration. Additionally, in patients who progressed to ARDS, the hypercatabolic state and proinflammatory response might exacerbate malnutrition, creating a vicious cycle of deteriorating respiratory function [44]. The integration of early, targeted nutritional support is essential in preventing these complications, which can help to modulate the systemic inflammatory response, preserve respiratory muscle strength, and reduce the risk and severity of ARDS.

Many studies have shown that malnutrition is strongly associated with poorer survival outcomes in gastrointestinal and pancreatic cancer [45,46], as well as in patients undergoing PD. In our study, patients with severe malnourishment had a more than threefold increase in 1-year mortality. However, the long-term survival rate in patients with pancreatic and other periampullary cancer was not statistically significant. This could be owing to the relatively small sample size, incomplete time surveillance of our study, and the overwhelming impact of cancer prognosis itself. We believe that a larger sample size would allow for better assessment of survival time and might signify severe malnourishment as an unfavorable prognosis factor.

There are several limitations to our study. First, the GNRI is a simple, practical tool that uses serum albumin and BMI for quick assessments. However, it overlooks key factors such as muscle mass, inflammation, and psychosocial aspects, limiting its ability to provide a comprehensive evaluation of nutritional status. Additionally, we did not evaluate our results using other widely recognized nutritional assessment tools such as the PNI, SGA, MNA, or skeletal muscle index. As a result, we cannot determine whether the GNRI is superior to these other nutritional assessment indicators based on our study alone. Second, since this was a retrospective, single-center study with a relatively small sample size, we were unable to separately analyze survival data across different periampullary cancer types. The small sample size and high heterogeneity in tumor types likely contributed to the lack of statistically significant differences in both tumor size and malnourishment groups. Third, the minimal impact of the ΔGNRI may be attributed to short-term changes in the GNRI following surgery, which might not adequately reflect prognosis and require long-term follow-up. Additionally, since the high-risk group was already in poor condition pre-surgery, the postoperative ΔGNRI generally shows improvement, potentially affecting the analysis. Further prospective, large-scale studies are necessary to evaluate the role of the GNRI and its influence on long-term clinical outcomes.

## 5. Conclusions

Our study demonstrated that a high preoperative GNRI risk is a significant predictor of adverse early postoperative outcomes including major complications, PPCs, and delayed postoperative recovery in older adult patients with periampullary cancer who underwent pancreatoduodenectomy for periampullary cancer. The increased 1-year mortality highlights the need for careful preoperative assessment and, potentially, alternative treatment strategies in this severe malnourished population. Given the high early postoperative adverse effects, patients with severe malnourishment should be considered as presenting with conditionally borderline resectable status. These patients who are undergoing upfront surgery should be receiving aggressive nutrition intervention with carefully preoperative safety evaluation owing to their lower survival benefit and the increased risk of early mortality and 1-year mortality.

## Figures and Tables

**Table 1:** Initial attributes of patients undergoing pancreatoduodenectomy classified by GNRI categories. (*n* = 199).

		Total (*n* = 199)	Severely Malnourished (*n* = 19)	Moderately/Mildly Malnourished (*n* = 109)	No Malnourishment (*n* = 71)	***p*-Value**
Age (years), median (IQR)		72 (8)	76.0 (11)	73.0 (9)	71.0 (6)	0.006
Male gender, *n* (%)		169 (56.1%)	11 (57.9%)	56 (51.4%)	47 (66.2%)	0.145
BMI (kg/m^2^), median (IQR)		22.93 (4.60)	19.01 (4.33)	22.78 (5.02)	24.14 (3.71)	<0.001
Abdominal surgery history, *n* (%)		42 (21.1%)	5 (26.3%)	21 (19.3%)	16 (22.5%)	0.734
Hypertension, *n* (%)		115 (57.8%)	6 (31.6%)	68 (62.4%)	41 (57.7%)	0.043
Diabetes mellitus, *n* (%)		69 (34.7%)	2 (10.5%)	35 (32.1%)	32 (45.1%)	0.014
Chronic kidney disease, *n* (%)		20 (10.1%)	3 (15.8%)	11 (10.1%)	6 (8.5%)	0.640
Coronary heart disease, *n* (%)		40 (20.1%)	5 (26.3%)	17 (15.6%)	18 (25.4%)	0.217
Chronic obstructive pulmonary disease, *n* (%)		9 (4.5%)	3 (15.8%)	4 (3.7%)	2 (2.8%)	0.083
ASA physical status, *n* (%)	1	4 (2.0%)	0	2 (1.8%)	2 (2.8%)	0.244
	2	48 (24.1%)	3 (15.8%)	23 (21.1%)	22 (31.0%)	
	3	145 (72.9%)	16 (84.2%)	84 (77.1%)	45 (63.4%)	
	4	2 (1.0%)	0	0	2 (2.8%)	
ECOG, *n* (%)	0	180 (90.5%)	13 (68.4%)	98 (89.9%)	69 (97.2%)	0.002
	1	16 (8.0%)	6 (31.6%)	8 (7.3%)	2 (2.8%)	
	2	3 (1.5%)	0	3 (2.8%)	0	
Tumor Type, *n* (%)	Ampullary cancer	57 (28.6%)	7 (36.8%)	32 (29.4%)	18 (25.4%)	0.943
	CBD cancer	41 (20.6%)	3 (15.8%)	25 (22.9%)	13 (18.3%)	
	Pancreatic cancer	85 (42.7%)	8 (42.1%)	43 (39.4%)	34 (47.9%)	
	NEC	6 (3.0%)	0	3 (2.8%)	3 (4.2%)	
	Duodenal cancer	10 (5.0%)	1 (5.3%)	6 (5.5%)	3 (4.2%)	
Operation Type, *n* (%)	Open PD	157 (78.9%)	17 (89.5%)	82 (75.2%)	58 (81.7%)	0.288
	Minimal invasive PD	42 (21.1%)	2 (10.5%)	27 (24.8%)	13 (18.3%)	

IQR, interquartile range; BMI, body mass index; ASA, American Society of Anesthesiologists; ECOG, Eastern Cooperative Oncology Group; GNRI, Geriatric Nutritional Risk Index; CBD, common bile duct; NEC, neuroendocrine carcinoma. Severely malnourished (GNRI ≤ 82), moderately/mildly malnourished (82 < GNRI ≤ 98), and no malnourishment (GNRI > 98) groups.

**Table 2:** The early postoperative outcomes of patients who received pancreatoduodenectomy classified by the GNRI. (*n* = 199).

		Total (*n* = 199)	Severely Malnourished (*n* = 19)	Moderately/Mildly Malnourished (*n* = 109)	No Malnourishment (*n* = 71)	*p*-Value
Operative time (minutes), median (IQR)		469.0 (192.0)	596.0 (294.0)	450.0 (153.0)	480.0 (200.0)	0.023
Estimated blood loss (mL), mean ± SD		358.5 ± 470.6	415.8 ± 334.5	345.6 ± 377.3	438.8 ± 610.3	0.188
Tumor size (cm), mean ± SD		3.0 ± 1.5	3.4 ± 1.4	2.9 ± 1.5	3.3 ± 1.6	0.024
ΔGNRI%, median (IQR)		4.5 (15.5)	−5.9 (16.4)	2.5 (12.3)	11.4 (12.3)	<0.001
Postoperative stays (days), median (IQR)		25.0 (14)	33.0 (17.0)	26.0 (16)	22.0 (11.0)	<0.001
Length of ICU stay (days), mean ± SD		7.8 ± 15.8	8.7 ± 11.3	9.4 ± 20.1	5.1 ± 6.2	0.152
Soft diet (days), mean ± SD		10.5 ± 12.7	8.8 ± 3.5	11.2 ± 15.5	9.8 ± 8.9	0.561
Ambulation (days), median (IQR)		6.0 (3.0)	8.0 (11.0)	6.0 (4.0)	6.0 (2.0)	0.044
TPN use (days), mean ± SD		12.3 ± 12.4	14.5 ± 18.1	13.2 ± 12.7	10.4 ± 9.6	0.150
Drain remove (days), median (IQR)		21.0 (12.0)	23.0 (15)	22.0 (13.0)	20.0 (10.0)	0.039
Grade 3b complication, *n* (%)		42 (21.1%)	8 (42.1%)	24 (22.0%)	10 (14.1%)	0.027
Pancreatic fistula, *n* (%)	0	118 (59.3%)	15 (78.9%)	62 (56.9%)	41 (57.7%)	0.372
	1	48 (24.1%)	3 (15.8%)	27 (24.8%)	18 (25.4%)	
	2	21 (10.6%)	1 (5.3%)	10 (9.2%)	10 (14.1%)	
	3	12 (6.0%)	0	10 (9.2%)	2 (2.8%)	
Postoperative pulmonary complication, *n* (%)		21 (10.6%)	5 (26.3%)	13 (11.9%)	3 (4.2%)	0.016
Surgical site infection, *n* (%)		71 (35.7%)	8 (42.1%)	44 (40.4%)	19 (26.8%)	0.146
Postoperative bleeding, *n* (%)		17 (8.5%)	1 (5.3%)	11 (10.1%)	5 (7.0%)	0.670
Delay gastric emptying, *n* (%)		42 (21.1%)	7 (36.8%)	20 (18.3%)	15 (21.1%)	0.190
30-day mortality, *n* (%)		5 (2.5%)	0	3 (2.8%)	2 (2.8%)	1.000
1-year mortality, *n* (%)		101 (50.8%)	15 (78.9%)	57 (52.3%)	29 (40.8%)	0.011

IQR, interquartile range; SD, standard deviation; ICU, Intensive care unit; TPN, Total parenteral nutrition. Severely malnourished (GNRI ≤ 82), moderately/mildly malnourished (82 < GNRI ≤ 98), and no malnourished (GNRI > 98) groups. ΔGNRI% = (preoperative GNRI − postoperative GNRI)/preoperative GNRI × 100%.

**Table 3:** Univariate and multivariate analysis of factors associated with significant postoperative complications (Clavien–Dindo grade ≥ IIIb).

Variables	Unadjusted OR (95% CI)	*p*-Value	Adjusted OR (95% CI)	*p*-Value
Age	1.04 (0.98–1.10)	0.219	—	
Sex (Male)	1.45 (0.72–2.93)	0.303	—	
BMI	1.00 (0.92–1.10)	0.848	—	
Hypertension	1.24 (0.62–2.5)	0.544	—	
Diabetes mellitus	0.81 (0.39–1.68)	0.569	—	
ASA physical status				
I–II	Reference		Reference	
III–V	4.18 (1.41–12.38)	0.010	3.56 (1.18–10.78)	0.025
GNRI score			—	
No malnourishment	Reference		Reference	
Moderately/mildly malnourished	1.72 (0.77–3.86)	0.448	1.71 (0.74–3.96)	0.210
Severely malnourished	4.43 (1.43–13.73)	0.010	3.44 (1.06–11.22)	0.040
MIS approach ^†^	0.70 (0.29–1.70)	0.429	—	
Operative time				
Time < 480 min	Reference		Reference	
Time > 480 min	2.02 (1.01–4.06)	0.048	1.63 (0.77–3.43)	0.202
Estimated blood loss				
<400 mL	Reference		—	
>400 mL	2.05 (1.01–4.16)	0.046	1.87 (0.89–3.95)	0.101

^†^ Compared to open approach. OR, odds ratio; CI, confidence interval; BMI, body mass index; ASA, American Society of Anesthesiologists; GNRI, Geriatric Nutritional Risk Index; MIS, minimal invasive surgery. Severely malnourished (GNRI ≤ 82), moderately/mildly malnourished (82 < GNRI ≤ 98), and no malnourishment (GNRI > 98) groups.

**Table 4:** Univariate and multivariate analysis of factors associated with postoperative pulmonary complications.

Variables	Unadjusted OR (95% CI)	*p*-Value	Adjusted OR (95% CI)	*p*-Value
Age	1.09 (1.01–1.19)	0.038	1.06 (0.98–1.15)	0.159
Sex (Male)	3.55 (1.15–10.97)	0.028	4.40 (1.37–14.15)	0.013
BMI	1.00 (0.88–1.13)	0.996	—	
Hypertension	1.53 (0.59–3.96)	0.386	—	
Diabetes mellitus	0.283 (0.08–0.99)	0.049	0.37 (0.10–1.36)	0.134
ASA physical status				
I–II	Reference		—	
III–V	3.71 (0.83–16.52)	0.085	—	
GNRI score				
No malnourishment	Reference		Reference	
Moderately/mildly malnourished	3.07 (0.84–11.19)	0.089	3.04 (0.80–11.57)	0.102
Severely malnourished	8.10 (1.73–37.86)	0.008	5.91 (1.15–30.47)	0.034
MIS approach ^†^	0.59 (0.17–2.12)	0.423	—	
Operative time				
Time < 480 min	Reference		—	
Time > 480 min	2.34 (0.90–6.08)	0.080	—	
Estimated blood loss				
<400 mL	Reference		—	
>400 mL	1.87 (0.74–4.70)	0.185	—	

^†^ Compared to open approach. OR, odds ratio; CI, confidence interval; BMI, body mass index; ASA, American Society of Anesthesiologists; GNRI, Geriatric Nutritional Risk Index; MIS, minimal invasive surgery. Severely malnourished (GNRI ≤ 82), moderately/mildly malnourished (82 < GNRI ≤ 98), and no malnourishment (GNRI > 98) groups.

**Table 5:** Univariate and multivariate analysis of factors associated with extended hospital stays after surgery (>28 days).

Variables	Unadjusted OR (95% CI)	*p*-Value	Adjusted OR (95% CI)	*p*-Value
Age	1.06 (1.04–1.11)	0.035	1.05 (0.99–1.11)	0.118
Sex (Male)	1.31 (0.74–2.34)	0.354	—	
BMI	0.98 (0.90–1.06)	0.541	—	
Hypertension	1.16 (0.65–2.1)	0.605	—	
Diabetes mellitus	0.851 (0.47–1.55)	0.598	—	
ASA physical status				
I–II	Reference		—	
III–V	1.74 (0.89–3.40)	0.109	—	
GNRI score				
No malnourished	Reference		Reference	
Moderate/mild malnourished	2.01 (1.06–3.81)	0.033	1.96 (1.01–3.80)	0.047
Severe malnourished	4.37 (1.51–12.69)	0.007	3.54 (1.18–10.59)	0.024
MIS approach ^†^	0.45 (0.21–0.96)	0.040	0.42 (0.19–0.92)	0.030
Operative time				
Time < 480 min	Reference		—	
Time > 480 min	1.71 (0.97–3.04)	0.065	—	
Estimated blood loss				
<400 mL	Reference		—	
>400 mL	1.33 (0.72–2.45)	0.365	—	

^†^ Compared to open approach. OR, odds ratio; CI, confidence interval; BMI, body mass index; ASA, American Society of Anesthesiologists; GNRI, Geriatric Nutritional Risk Index; MIS, minimal invasive surgery. Severely malnourished (GNRI ≤ 82), moderately/mildly malnourished (82 < GNRI ≤ 98), and no malnourishment (GNRI > 98) groups.

**Table 6:** Univariate and multivariate analysis of factors associated with postoperative one-year early mortality.

Variables	Unadjusted OR (95% CI)	*p*-Value	Adjusted OR (95% CI)	*p*-Value
Age	1.03 (0.98–1.09)	0.286	—	
Sex (Male)	1.19 (0.68–2.09)	0.540	—	
BMI	0.91 (0.84–0.99)	0.022	0.96 (0.88–1.04)	0.287
Hypertension	0.70 (0.40–1.23)	0.211	—	
Diabetes mellitus	0.49 (0.27–0.88)	0.018	0.59 (0.32–1.09)	0.092
ASA physical status				
I–II	Reference		—	
III–V	1.16 (0.61–2.18)	0.653	—	
GNRI score				
No malnourishment	Reference		Reference	
Moderately/mildly malnourished	1.59 (0.87–2.90)	0.134	1.41 (0.75–2.62)	0.284
Severely malnourished	5.43 (1.64–18.04)	0.006	3.65 (1.01–13.13)	0.048
MIS approach ^†^	1.09 (0.55–2.15)	0.812	—	
Operative time				
Time < 480 min	Reference		—	
Time > 480 min	0.87 (0.50–1.52)	0.625	—	
Estimated blood loss				
<400 mL	Reference		—	
>400 mL	1.55 (0.84–2.86)	0.161	—	

^†^ Compared to open approach. OR, odds ratio; CI, confidence interval; BMI, body mass index; ASA, American Society of Anesthesiologists; GNRI, Geriatric Nutritional Risk Index; MIS, minimal invasive surgery. Severely malnourished (GNRI ≤ 82), moderately/mildly malnourished (82 < GNRI ≤ 98), and no malnourishment (GNRI > 98).

## Data Availability

The raw data supporting the conclusions of this article will be made available by the corresponding author, S.-M.Y., upon reasonable request.

## Supplementary Materials

The following supporting information can be downloaded at: https://www.mdpi.com/article/10.3390/jcm14020655/s1, Table S1: Early postoperative outcomes of patients who received pancreatoduodenectomy classified by ΔGNRI. (*n* = 199).

## Abbreviations

Pancreaticoduodenectomy (PD), Mini Nutritional Assessment (MNA), Subjective Global Assessment (SGA), Geriatric Nutritional Risk Index (GNRI), acute respiratory distress syndrome (ARDS), ideal body weight (IBW), intensive care unit (ICU), postoperative pancreatic fistula (POPF), postoperative pulmonary complications (PPCs), overall survival (OS), odds ratio (OR), the American Society of Anesthesiologists (ASA), and the Prognostic Nutritional Index (PNI).
