# Supplementary material for: High Geriatric Nutritional Risk Index Risk as a Predictor of Postoperative Complications and Early Mortality in Older Adult Patients Undergoing Pancreatoduodenectomy for Periampullary Malignancies
Source: J Clin Med. 2025 Jan 20;14(2):655. doi: 10.3390/jcm14020655 (PMC11766299; doi:10.3390/jcm14020655)
Supplement: Supplementary file 1 [file jcm-14-00655-s001.zip › jcm-3359633-supplementary.pdf]

Supplementary Table S1. *Early postoperative outcomes of patients who received pancreatoduodenectomy classified by ΔGNRI. (n=199)*

	Total (*n*=199)	ΔGNRI<4.5% (*n*=101)	ΔGNRI>4.5% (*n*=98)	P-value
Operative time (minutes), median (IQR)	469.0 (192.0)	450.0 (210.0)	480.0 (200.0)	0.904
Estimated blood loss (ml), mean ± SD	358.5 ± 470.6	374.1 ± 365.1	356.9 ± 424.5	0.352
Tumor size (cm) , mean ± SD	3.0 ± 1.5	2.9 ± 1.3	3.3 ± 1.7	0.108
Postoperative stays (days), median (IQR)	25.0 (14)	25.0 (14.0)	24.0 (15)	0.301
Length of ICU stay (days) , mean ± SD	7.8 ± 15.8	10.8 ± 21.8	5.3 ± 6.1	0.154
Soft diet (days) , mean ± SD	10.5 ± 12.7	11.0 ± 16.0	9.9 ± 8.1	0.812
Ambulation (days), median (IQR)	6.0 (3.0)	7.0 (5.0)	6.0 (2.0)	0.062
TPN use (days) , mean ± SD	12.3 ± 12.4	13.8 ± 12.9	11.1 ± 12.2	0.003
Drain remove (days), median (IQR)	21.0 (12.0)	22.0 (11.0)	21.0 (13.0)	0.256
Grade 3b complication, *n* (%)	42 (21.1%)	8 (42.1%)	24 (22.0%)	0.201
Pancreatic fistula, *n* (%) 0	118 (59.3%)	60 (59.4%)	58 (59.2%)	0.333
1	48 (24.1%)	27 (26.7%)	21 (21.4%)	
2	21 (10.6%)	7 (6.9%)	14 (14.3%)	
3	12 (6.0%)	7 (6.9%)	5 (5.1%)	
Postoperative pulmonary complication, *n* (%)	21 (10.6%)	15 (14.9%)	6 (6.1%)	0.045
Surgical site infection, *n* (%)	71 (35.7%)	37 (36.6%)	34 (34.7%)	0.775
Postoperative bleeding, *n* (%)	17 (8.5%)	7 (6.9%)	10 (10.2%)	0.409
Delay gastric emptying, *n* (%)	42 (21.1%)	20 (19.8%)	22 (22.4%)	0.190
30-day mortality, *n* (%)	5 (2.5%)	3 (3.0%)	2 (2.0%)	1.000
1-Year mortality, *n* (%)	101 (50.8%)	46 (45.5%)	55 (56.1%)	0.136

IQR, interquartile range; SD, standard deviation; ICU, Intensive care unit; TPN, Total parenteral nutrition.

ΔGNRI% = (preoperative GNRI – postoperative GNRI) / preoperative GNRI × 100%
